# HyperSight CBCT image quality and metal artifact reduction for adaptive head and neck radiotherapy: Results from a prospective clinical trial

**DOI:** 10.1002/acm2.70338

**Published:** 2025-11-29

**Authors:** Abby Yashayaeva, R. Lee MacDonald, Kenny Zhan, Jennifer DeGiobbi, Natasha McMaster, Dave McAloney, Lucy Ward, Cheryl Anderson, Marc LeBlanc, Lara Best, Murali Rajaraman, Derek Wilke, Amanda Cherpak

**Affiliations:** ^1^ Department of Physics and Atmospheric Sciences Dalhousie University Halifax Nova Scotia Canada; ^2^ Department of Radiation Oncology, QE2 Cancer Centre Nova Scotia Health Halifax Nova Scotia Canada; ^3^ Department of Radiation Oncology Dalhousie University Halifax Nova Scotia Canada

**Keywords:** adaptive radiotherapy, cone beam computed tomography, metal artifact reduction

## Abstract

**Background:**

Accurate Hounsfield units (HU) are critical for dose calculation and anatomical visualization, but are often affected by dental artifacts in head and neck (H&N) cancer patients. The HyperSight cone‐beam computed tomography (CBCT) platform provides improved image quality over previous CBCT platforms and offers metal artifact reduction (iCBCT MAR) reconstruction.

**Purpose:**

This study evaluates the quality of HyperSight CBCT images compared to current clinical standards: TrueBeam CBCT for image guidance and fan‐beam CT (FBCT) from a CT simulator for treatment planning, using images captured during H&N cancer treatment.

**Methods:**

Images for 30 H&N cancer patients were acquired on a HyperSight CBCT, conventional TrueBeam and FBCT, with 24 patients exhibiting metal dental artifacts. The HyperSight images were reconstructed using iCBCT MAR and iterative (iCBCT Acuros) algorithms. The four image sets were rigidly registered and compared using the artifact index (AI) measured in the oral cavity and the percentile range (PR) measured in the oral cavity, brain, brainstem and eyes to assess image non‐uniformity. The HU accuracy was calculated relative to FBCT (baseline) for soft tissues (oral cavity, brainstem, submandibular and parotid glands), and bone (mandible). The contrast relative to baseline was evaluated between the oral cavity and nearby structures. Image‐based metrics were computed relative to FBCT including structural similarity index measure (SSIM), mean‐square error (MSE) and peak signal‐to‐noise ratio (PSNR).

**Results:**

The HyperSight iCBCT MAR images showed a significant reduction in AI values compared to the other images (*p* < 0.0004), but higher PR values indicating decreased HU uniformity compared to HyperSight iCBCT Acuros and FBCT (*p* < 0.0002). The soft‐tissue HU and contrast values were significantly closer to baseline in both HyperSight images compared to TrueBeam (HU: *p* < 0.001, contrast: *p* < 0.001). For soft‐tissue the HU mean absolute deviation (MAD) from baseline was 16 ± 10 HU for HyperSight iCBCT Acuros, 15 ± 10 HU for HyperSight iCBCT MAR, and 35 ± 22 HU for TrueBeam. For bone, the HU MAD from baseline was 153 ± 233 HU, 185 ± 268 HU, and 214 ± 212 HU, respectively. The HyperSight iCBCT Acuros algorithm achieved significantly superior SSIM, MSE, and PSNR metrics compared to TrueBeam and HyperSight iCBCT MAR in regions with large amounts of bone and air.

**Conclusions:**

HyperSight iCBCT MAR significantly reduced artifacts compared to HyperSight iCBCT Acuros, TrueBeam and FBCT, making it particularly beneficial for patients with metal implants. Both HyperSight reconstructions demonstrated improved soft‐tissue HU accuracy and contrast compared to TrueBeam, however the iCBCT Acuros algorithm may be preferred when metal‐induced artifacts are not a concern. These results support the suitability of HyperSight images in adaptive treatment workflows requiring accurate image quality, even with severe metal artifacts.

## 1 INTRODUCTION

The integration of cone‐beam CT (CBCT) in radiotherapy treatment machines has been essential for image‐guidance to ensure accurate patient positioning prior to dose delivery.^1^, ^2^ The CBCT image is used to align the patient to a treatment planning image which has been typically acquired on a fan‐beam CT (FBCT) scanner 1–2 weeks prior to the start of treatment. Recently, CBCT imaging has also become integral to the workflow for online adaptive approaches. By altering the treatment plan to account for daily changes that may occur in patient or tumor anatomy, online adaptive radiotherapy aims to enhance the overall effectiveness of therapy by reducing the dose to the organs at risk (OAR) and associated toxicities, while maintaining target coverage.^3^, ^4^ Head and neck cancer patients undergoing radiation therapy often experience significant anatomic changes, including weight loss and tumor shrinkage and may require a second planning CT scan and the creation of a new treatment plan.^5^, ^6^

While there have been improvements in CBCT image quality (e.g., through iterative reconstruction methods),^7^, ^8^ CBCT images have historically had inferior image quality compared to FBCT.^9^ This deficiency stems from factors like increased photon scattering due to the wide cone‐beam angle^10^ leading to artifacts, non‐uniformities, and reduced Hounsfield unit (HU) accuracy. While CBCT images are generally effective for matching daily anatomy, the image quality and HU accuracy offered by most on‐board imagers are unsuitable for the tumor and organ delineation and dose calculations necessary for treatment plan adaptation.^11^, ^12^ In order to retrieve adequate HU values on CBCT image data for dose calculations, often a synthetic CT (sCT) is created by deforming FBCT data onto CBCT data.^13^ However, uncertainties in the deformable image registration along with discrepancies in the HU reported for the same density tissue between the FBCT and CBCT can lead to an unrealistic sCT, and therefore inaccurate data for dose calculation.^14^, ^15^

As the clinical implementation of online adaptive radiotherapy continues, there is an increasing demand for CBCT scanners that provide image quality comparable to that of a FBCT. In more complex cases involving metallic implants, such as dental fillings, wires, screws, spine implants, joint prostheses, surgical clips, coils, and cardiac electronic devices, metal artifacts can be introduced during CT reconstruction.^16^, ^17^, ^18^, ^19^ These artifacts can distort HU values and obscure surrounding anatomy, potentially leading to inaccurate tumor or organ delineation and dose miscalculations.^20^, ^21^ This could result in underdosing of the tumor and overdosing of nearby organs, compromising treatment effectiveness and safety. To mitigate these issues, metal artifact reduction (MAR) reconstruction algorithms have been developed to enhance image quality and reduce distortion in CT scans for patients with metal prostheses or implants, improving overall treatment accuracy.^22^

The HyperSight imaging system on the Ethos radiotherapy platform (Varian Medical Systems, Palo Alto, CA) is equipped with an 86 cm × 43 cm detector that features a novel cesium iodide (CsI) scintillator, a scatter‐reducing grid, low‐noise electronics, and a fast image readout (up to 70 frames per second),^23^ providing improved dose efficiency and clearer images compared to previous generations. HyperSight offers advanced features, such as a reduced acquisition time of just 6 s, an extended field of view (eFoV) of 70 cm and various image reconstruction methods, including Feldkamp Davis Kress (FDK) back‐projection, iCBCT, iCBCT Acuros, and iCBCT MAR. Varian's iCBCT with Acuros mode incorporates scatter correction and is recommended by the manufacturer for achieving the highest accuracy of HU values, enhancing image quality and improving dose calculation precision.^24^, ^25^ HyperSight has been shown to produce CBCT images with image quality superior to previous CBCT platforms^23^, ^26^ and HU accuracy comparable to standard FBCT images.^27^, ^28^ Furthermore, evaluations of MAR^29^ and eFoV^30^ implemented on HyperSight have shown promising results when tested on tissue‐equivalent phantoms. The HyperSight imaging platform has the potential to facilitate direct dose calculations, thereby eliminating the need for a sCT within the Ethos adaptive radiotherapy system, as well as reducing the necessity for an additional CT simulation for offline re‐planning. The current workflow for re‐planning after treatment begins requires a repeat CT appointment which puts a strain on hospital resources, as planning and quality assurance are prioritized to minimize disruption to the patient's treatment delivery, and also adds burden to the patient by requiring extra appointments and imaging.

A thorough evaluation of HyperSight's imaging performance and advanced features using real patient data, and benchmarking against the current standards for image‐guidance and treatment planning, is critical before confidently integrating this technology into routine clinical practice. In this investigation, we assess the quality of images acquired on HyperSight using iCBCT Acuros and iCBCT MAR reconstruction modalities, compared to those acquired on a TrueBeam CBCT (Varian Medical Systems) and FBCT from a CT simulator (CTsim, GE Optima). Key quantitative factors include the HU accuracy and contrast of various head and neck OARs, the severity of artifacts produced by dental materials in the oral cavity, and image non‐uniformity in the brain. This patient‐study involves data for 30 participants and presents the first large‐population image quality results for patients with head and neck cancers receiving radiation treatment on the HyperSight imaging platform.

## 2 METHODS

### 2.1 Ethical approval and participants

The prospective study was registered under ClinicalTrials.gov on July 07, 2023, with the identifier NCT05666193 and received approval from the Research Ethics Board on February 6, 2023, under REB File#: 1028935. Patients aged 19 years or older who were receiving radiation therapy for head and neck cancer on a TrueBeam platform with a dose of 70 Gy over 35 daily fractions were eligible for this study. A total of 30 patients were enrolled in the study, with recruitment and data collection for the cohort taking place from August 2023 to October 2024. Among this cohort, 22 patients had oropharyngeal tumors, two had tonsillar tumors, one had a hypopharyngeal tumor, one had a nasopharyngeal tumor, and four had laryngeal involvement. Each participant signed an informed consent document and was assigned a unique identifier to ensure the anonymization of all collected data.^31^

### 2.2 Image acquisition

Patients were set up on the treatment couch in treatment position with a thermoplastic immobilization mask before image acquisition. Scans were acquired on a CTsim FBCT that did not have MAR available at the time of acquisition, and a conventional TrueBeam (version 2.7 MR4) CBCT with FDK reconstruction as part of routine clinical practice: for treatment planning and patient positioning, respectively. HyperSight CBCT scans were acquired on an Ethos radiotherapy platform, with the longitudinal imaging field of view (FOV) extending from the top of the head to 38 cm into the lungs, using the iCBCT Acuros reconstruction mode on the same day as the CTsim FBCT, which was obtained during the patient's simulation process. The TrueBeam CBCT scans were acquired on the first day of treatment. Elapsed time between these 2 days ranged from 12 to 20 days. HyperSight and TrueBeam CBCTs were also acquired on the day of the patients’ 21st treatment fraction for the clinical trial as part of a broader evaluation of the adaptive capabilities on Ethos. Since they were acquired weeks after the initial FBCT images, the anatomy and contours changed over time so we could not compare their image quality against the initial FBCT images, and for this reason the fraction 21 images were not included in the analysis. Analysis of anatomical changes over this time and the impact of plan adaptation will be the subject of future publications. The protocols used for image acquisition are summarized in Table 1.

**TABLE 1:** Imaging protocol parameters used for image acquisition in the study.

	CTsim FBCT	TrueBeam CBCT	HyperSight CBCT
Exposure (mAs)	32 – 69	150 – 151	174 – 350
X‐ray tube voltage (kVp)	120	100	125
Slice thickness (mm)	2.5	2	2
Pixel size (mm)	1.27	0.51 – 0.91	1.37
CTDIvol (mGy)	22 – 26	3.17 – 3.20	3.94 – 3.95
Axial FOV (mm)	67	26 – 46	31 – 72
Longitudinal FOV (mm)	45 – 52	19 – 34	38

### 2.3 Preprocessing

The raw HyperSight projection data was exported and reconstructed in the Varian Research Portal to generate a second HyperSight image using the iCBCT MAR reconstruction mode. All images were imported into the Eclipse treatment planning system (version 18, Varian Medical Systems) and aligned to the CTsim FBCT through rigid image registration. The treatment planning OAR structures were copied from the FBCT to the other image sets, and reviewed and adjusted by dosimetrists as needed to reflect any small differences in positioning. For all OARs, a 1 mm margin was contracted to reduce contour uncertainties near the boundary.

### 2.4 Image analysis

#### 2.4.1 ROI‐based metrics

The four image sets were compared based on the following ROI‐based metrics: artifact index, 90th–10th percentile range, contrast, and HU accuracy relative to CTsim FBCT.

#### 2.4.2 Artifact index

Images for 24 patients exhibited metal artifacts arising from dental implants and fillings, appearing as bright and dark streaks. These artifacts overlapped and obscured key anatomical structures, including the planning target volume (PTV) and oral cavity. The artifact index (AI), defined in Equation (1)^32^ is based on the standard deviation of pixel values in regions affected by metal artifacts compared to those in unaffected areas.

(1) $$AI=\sqrt{SD{\left(ROI_{artifact}\right)}^{2}-SD{\left(ROI_{background}\right)}^{2}}$$

**FIGURE 1:**
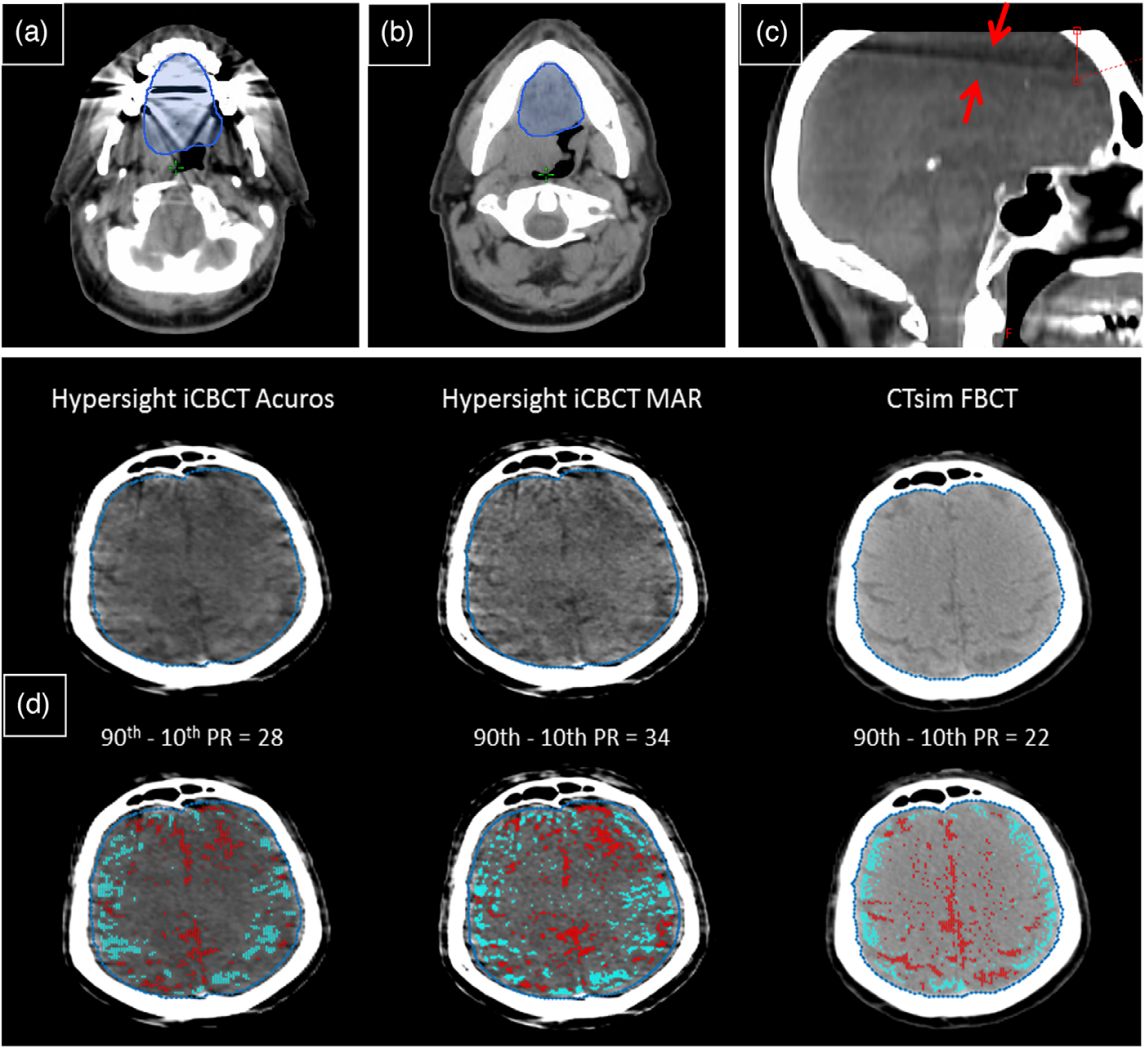
The slices used to define (a) the artifact ROI and (b) the background ROI, for calculating the artifact index. Both slices are from the same FBCT image of a single patient, with the oral cavity structure shown in blue. (c) Shows the cone‐beam effect artifact in the HyperSight iCBCT Acuros image, and (d) is the slice selected to evaluate the brain percentile range (PR), displayed with the same window width and level settings. The PR is recorded for this patient as an example, and the bottom row of (d) shows pixels with HU above the 90^th^ percentile in that slice colored in red, and pixels with HU below the 10^th^ percentile in teal.

The artifact region of interest ($ROI_{artifact}$) was defined in the oral cavity slice with the highest standard deviation of pixels ($SD(ROI_{artifact})$), while the background region of interest ($ROI_{background}$) was defined in the slice with the lowest standard deviation of pixels ($SD(ROI_{background})$). Figure 1a and b shows the artifact and background slices selected within the oral cavity in one patient's FBCT image, as a representative example. The artifact index can be dependent on ROI size, for example smaller ROIs may not fully capture the extent of an artifact or may be overly influenced by local fluctuations, while larger ROIs tend to average out these variations. Therefore, maintaining a consistent ROI size across scans and patients was important to ensure fair and comparable measurements. To determine the appropriate ROI size, we evaluated the AI by defining $ROI_{artifact}$ and $ROI_{background}$ as circles centered in the artifact slice and background slice of the oral cavity, respectively, with radii ranging from 2 to 30 mm in increments of 2 mm. Out of the 30 patients, three were excluded from the analysis due to the presence of mouthpieces, and six were excluded because they were edentulous without dental implants.

#### 2.4.3 HU accuracy relative to baseline

HU accuracy is critical for direct dose calculations on HyperSight images during adaptive radiotherapy on the Ethos platform. Assessing HU accuracy in real patient images accounts for the complexities in tissue composition that phantoms cannot fully replicate and ensures that the imaging system performs reliably under realistic conditions, for example patient movement. The mean HU was computed for various soft‐tissue OAR structures including the oral cavity, brainstem, and the left/right submandibular glands and parotid glands, as well as for bone, specifically the mandible. Measurements for the OARs were conducted across all four image sets.

The HU deviations from baseline were computed for each OAR, defined in Equation (2).

(2) $$\Delta HU_{\mathrm{OAR}}=\bar{HU_{I}}-\bar{HU_{B}}$$

where $\;\bar{HU_{I}}\;$ is the mean HU in the central slice of an OAR in image I (image under evaluation), and $\bar{HU_{B}}$ is the mean HU in the central slice of the same OAR in the baseline image B (CTsim FBCT). A 1 mm margin was excluded from the OAR contours to minimize HU uncertainty associated with the boundary. For the oral cavity, $\bar{HU_{I}}$ and $\bar{HU_{B}}$ were computed on the slice in the OAR with the lowest standard deviation, previously defined as $\;ROI_{background}$ for the AI metric, to avoid metal artifacts.

#### 2.4.4 Contrast

Contrast in patient images directly affects the visibility and differentiation of anatomical structures and is critical for accurate contouring. The contrast metric, defined by Equation (3), was used to quantify the clarity and differentiation between structures of different densities.

(3) $$Contrast=\left|\bar{HU_{OAR1}}\;-\bar{HU_{OAR2}}\right|$$

where $\bar{HU_{OAR1}}$ and $\bar{HU_{OAR2}}$ are the mean HU values of two OAR structures, computed on a slice with minimal metal artifacts present. This evaluation focused on nearby structures with similar HU levels, including the left parotid and oral cavity, right parotid and oral cavity, left submandibular gland and oral cavity, right submandibular gland and oral cavity. Contrast deviations relative to baseline (CTsim FBCT) were assessed.

#### 2.4.5 Percentile range

Image uniformity reflects the HU consistency across regions that should be homogeneous and can impacts the reliability of dose calculations in radiotherapy planning. The HU uniformity in OARs with homogenous tissue, including the oral cavity, brain, brainstem, and left and right eyes excluding the lens and retina, was quantified using the 90th–10th percentile range, defined in Equation (4).

(4) $$PR=P_{90}-P_{10}$$

where *P*_90_ is the 90^th^ percentile and *P*_10_ is the 10^th^ percentile of the pixels values within the ROI.

The brain and eyes were not within the field of view of the TrueBeam images, therefore the PR of these OARs was only evaluated for the CTsim FBCT and HyperSight images. In the HyperSight images, a peripheral V‐shaped artifact caused by the cone‐beam effect was observed, affecting the upper portion of the brain. This artifact typically extended 2.5 cm from the top of the image, shown in Figure 1c). Consequently, for the brain, the ROI used to evaluate the PR metric was defined as the brain structure on a slice located halfway between the base of the peripheral artifact and the top of the cerebral ventricles, shown in Figure 1d as an example for one patient. In the bottom row of Figure 1d, the pixels with HU above the 90th percentile are shown in red, and pixels with HU below the 10th percentile are shown in teal.

The PR is less sensitive to local anatomical variations compared to the peak deviation non‐uniformity metric, which is defined by the difference between maximum and minimum pixel values within the ROI.^33^ Despite this advantage, the percentile range may still be influenced by inherent anatomical variability, and capture real tissue density differences alongside image non‐uniformity. To further address this limitation and isolate the evaluation of image uniformity, the 90th–10th PR was also analyzed in the same location of the brain of an anthropomorphic phantom (ATOM Phantom Family, Adult Female, Sun Nuclear).

Each of these metrics were analyzed to assess the image quality and hence determine how accurately the HyperSight images can represent critical structures. For all metrics, the Wilcoxon signed‐rank test p‐values were used to compare the results across the four image sets. To determine accuracy of HU and contrast, the mean absolute deviations (MAD) from CTsim FBCT, averaged across all patients and OARs, was computed for these metrics.

#### 2.4.6 Image‐based metrics

The structural similarity index measure (SSIM), mean‐square error (MSE) and peak signal‐to‐noise ratio (PSNR) values were computed using the CTsim FBCT images as the reference. Since the CTsim FBCT images were not reconstructed with MAR, the analysis was restricted to two cropped regions; one spanning five slices inferior to the bottom of the oral cavity and the other spanning five slices superior to the top of the oral cavity, to minimize the influence of artifacts. Pixels corresponding to air outside the patient were excluded from the calculations.

#### 2.4.7 Structural similarity index measure

SSIM compares two images in terms of brightness (luminance), intensity variation (contrast), and pixel arrangement (structure), with a value of 1 indicating perfect similarity. It is expressed as:

(5) $$SSIM\;\left(x,y\right)=\frac{\left(2\mu_{x}\mu_{y}+C_{1}\right)\left(2\sigma_{xy}+C_{2}\right)}{\left(\mu_{x}^{2}+\mu_{y}^{2}+C_{1}\right)\left(\sigma_{x}^{2}+\sigma_{y}^{2}+C_{1}\right)}\;$$

where *μ_x_*, *μ_y_*, *σ_x_*, *σ_y_*, and *σ_xy_* are the means, standard deviations, and cross‐covariance for the image *x* and reference image *y*. By default, the SSIM function uses regularization constants *C*_1_ = (0.01*L)^2^ and *C*_2_ = (0.03*L)^2^ where *L* is the dynamic range among both images.

#### 2.4.8 Mean square error

MSE measures the average squared difference in pixel intensity between two images with lower values indicating greater similarity. It is defined as:

(7) $$MSE=\frac{1}{MN}\;\sum_{i=0}^{M-1}\sum_{j=0}^{N-1}{\left(x\left(i,j\right)-y\left(i,j\right)\right)}^{2}$$

Here, *M* and *N* are the dimensions of the images *x* and *y*.

#### 2.4.9 Peak signal‐to‐noise ratio

PSNR quantifies the ratio between the maximum intensity and the error relative to the reference image with higher values indicating higher resemblance and lower noise deviations. It is defined as:

(6) $$PSNR=\frac{10*\mathrm{lo}g_{10}\;\left(peakval^{2}\right)}{MSE}\;$$

where peakval is the maximum pixel value in the reference image data.

## 3 RESULTS

**FIGURE 2:**
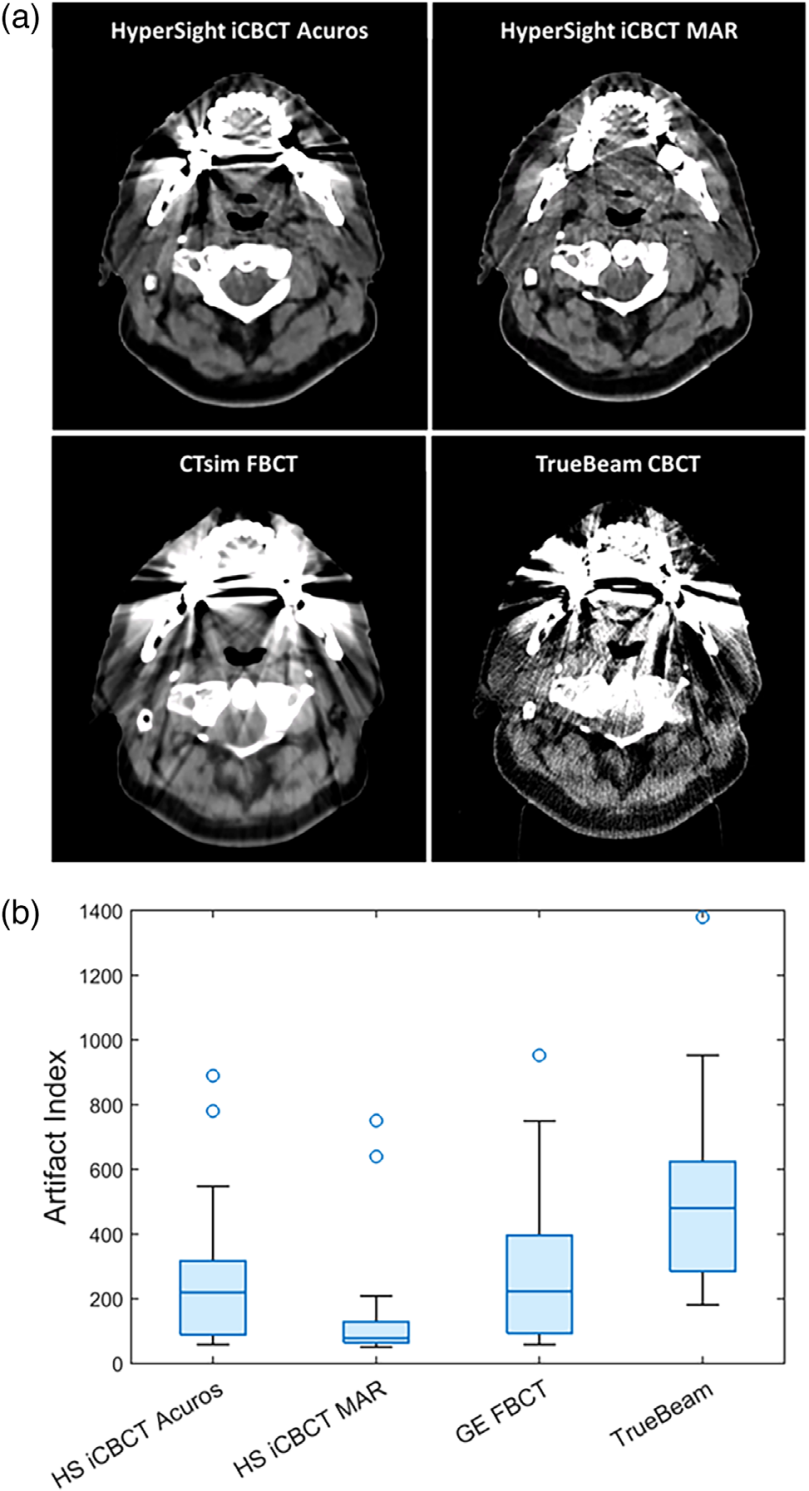
(a) Images highlighting metal artifacts in the oral cavity for one study patient using the same window width and level settings, and (b) the AI measured in the oral cavity for 24 patients who presented metal artifacts. The line inside of each box is the median, the top and bottom edges of each box correspond to the 0.75 and 0.25 quantiles, respectively, while the whiskers represent the maximum and minimum values. Outliers, displayed as circles, were calculated using 1.5 times the interquartile range (0.75–0.25).

Figure 2a shows images acquired on HyperSight iCBCT Acuros (HS iCBCT Acuros), HyperSight iCBCT MAR (HS iCBCT MAR), CTsim FBCT and TrueBeam CBCT imaging platforms for one patient from the study, as a representative example. The window level was fixed for all images (−270 HU to 550 HU). A visible reduction in metal artifact is observed for the HyperSight iCBCT MAR image.

### 3.1 ROI‐based metrics

The AI metric typically stabilized within a variation of 2%–8% for ROIs with radii greater than 16 mm among all patients and image types. To ensure a conservative approach, a circle centered in the oral cavity contour with a radius of 20 mm was chosen for the analysis. This effectively minimizes the dependence of the AI on the size of the ROI, allowing for a more consistent evaluation across different datasets. Figure 2b displays the artifact index computed in the oral cavity. The HyperSight iCBCT MAR images exhibited a significant reduction in artifact index values (*p* < 0.0004) compared to all other imaging modalities. Both HyperSight images and the CTsim FBCT image presented significantly lower artifact index values compared to TrueBeam (*p* < 0.0008), however HyperSight iCBCT Acuros and CTsim FBCT were not statistically different (*p* = 0.13).

**FIGURE 3:**
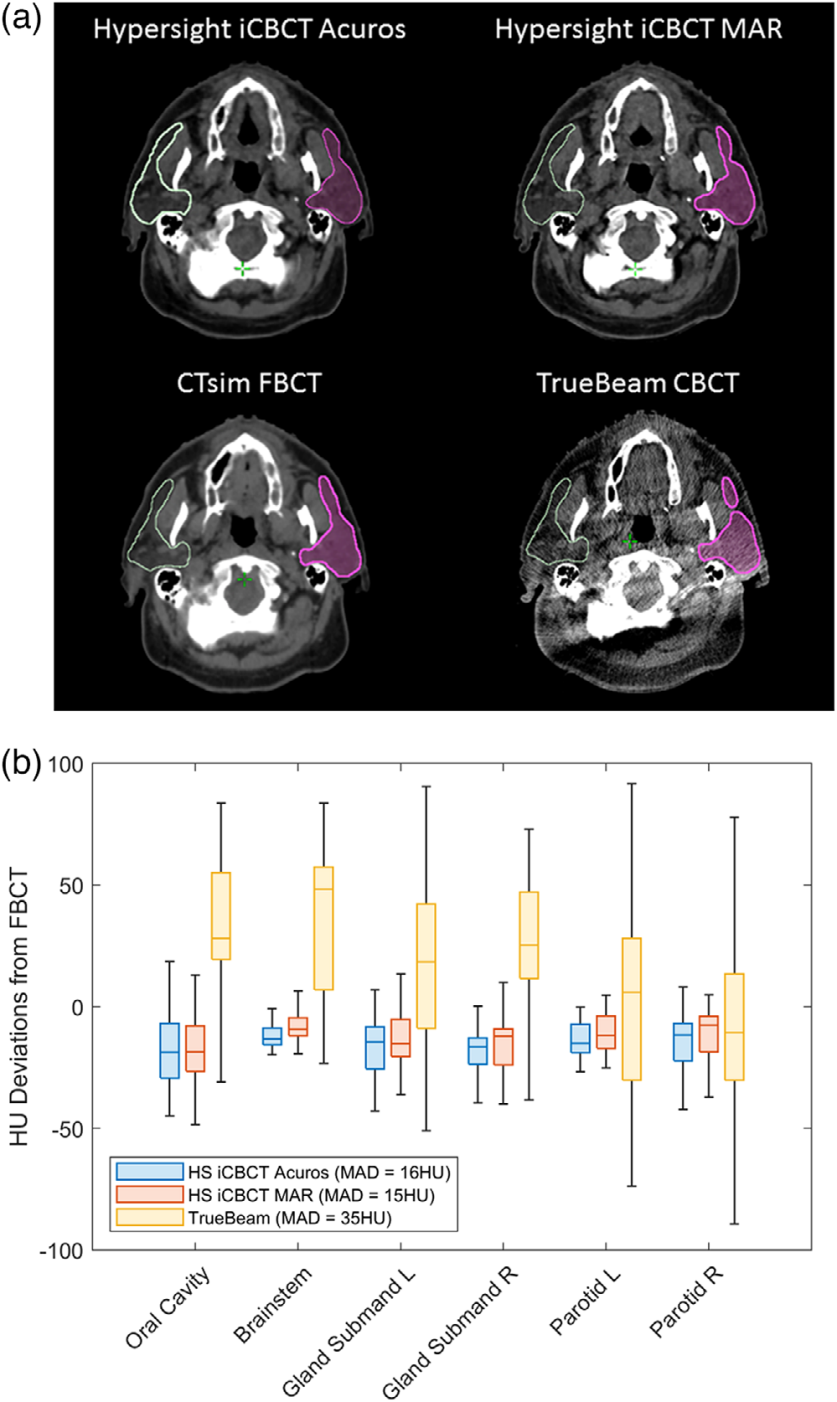
(a) Images showing the left and right parotid gland contours on the same slice for one study patient, using the same window width and level settings, and (b) the soft‐tissue HU deviations from FBCT for various OARs showing the median, 0.25 and 0.75 quantiles, and the maximum and minimum for all patients. The mean of absolute deviations from FBCT, averaged across all patients and OARs are displayed in the legend.

**FIGURE 4:**
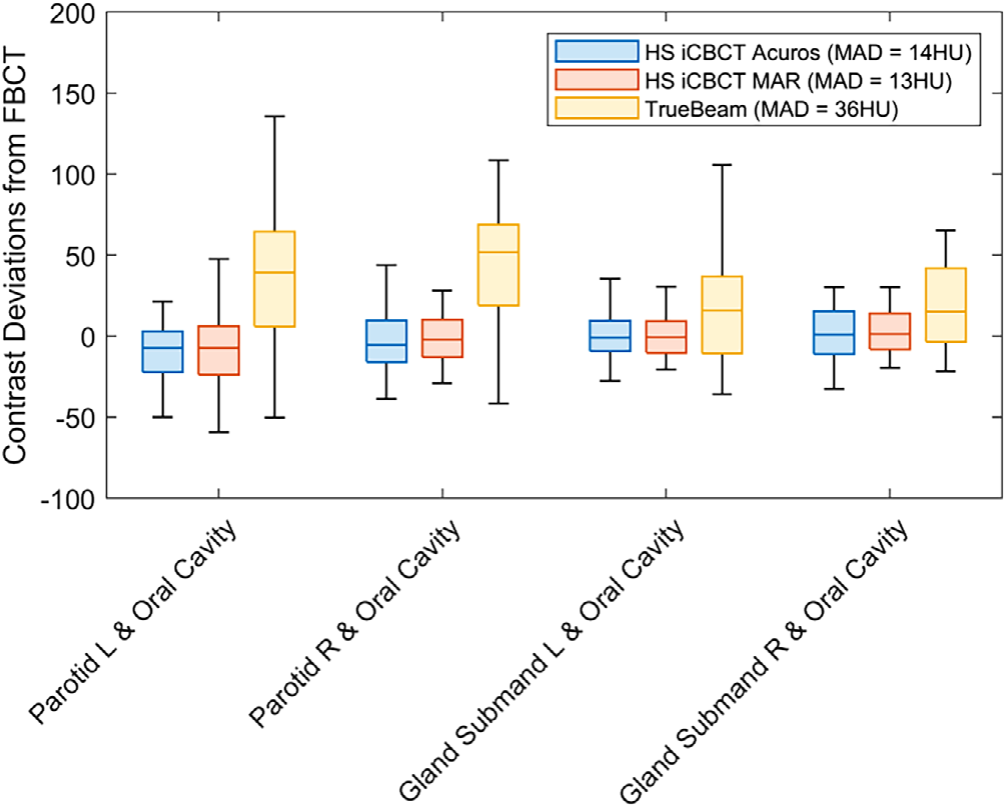
Contrast deviations from FBCT for various pairs of OARs showing the median, 0.25 and 0.75 quantiles, and the maximum and minimum for all patients. The mean of absolute deviations from FBCT, averaged across all patients and OARs are displayed in the legend.

Figure 3a shows contours of the left and right parotid glands on HyperSight iCBCT Acuros, HyperSight iCBCT MAR, CTsim FBCT and TrueBeam CBCT images for one patient from the study. The window level was fixed for all images (−150 HU to 615 HU). The image quality of the HyperSight iCBCT Acuros and HyperSight iCBCT MAR is comparable to the CTsim FBCT, allowing for easy visualization of the OARs, opposed to the TrueBeam CBCT. The soft‐tissue HU and contrast deviations from baseline are shown in Figures 3b and 4, respectively. The soft‐tissue HU MAD from baseline, averaged across patients and OARs, was lower for HyperSight iCBCT MAR (15 ± 10 HU) and iCBCT Acuros (16 ± 10 HU) compared to TrueBeam (35 ± 22 HU), with the uncertainty represented by the standard deviation. The contrast MAD from baseline was also the lowest for HyperSight iCBCT MAR (13 ± 12 HU) and iCBCT Acuros (14 ± 12 HU) compared to TrueBeam (36 ± 30 HU). The soft‐tissue HU and contrast values across patients were significantly closer to baseline in both HyperSight images compared to TrueBeam for the selected OARs (HU: *p* < 0.001, contrast: *p* < 0.001), except for HU measured in the parotid glands. The HyperSight images also demonstrated lower variability in the soft‐tissue HU and contrast deviations compared to TrueBeam. The mandible HU MAD from baseline, averaged across patients, was lower for iCBCT Acuros (153 ± 233 HU) and HyperSight iCBCT MAR (185 ± 268 HU) compared to TrueBeam (214 ± 212 HU), however the HyperSight images demonstrated greater variability.

**FIGURE 5:**
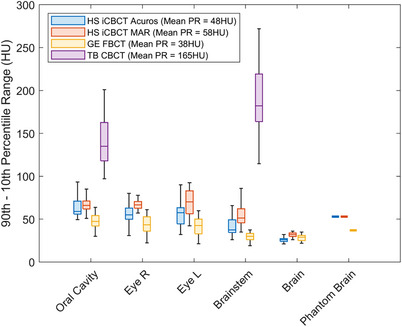
The percentile range of various OARs across 30 patients. The percentile range measured in the brain of an anthropomorphic phantom is also included. The line inside of each box is the median, the top and bottom edges of each box correspond to the 0.75 and 0.25 quantiles, respectively, while the whiskers represent the maximum and minimum values.

The 90^th^–10^th^ percentile range is displayed in Figure 5 for the oral cavity, eyes, brainstem and brain. The percentile ranges for the oral cavity and brainstem OARs, which were visible in the TrueBeam imaging field, were significantly higher in the TrueBeam images than the HyperSight iCBCT Acuros, MAR, and CTsim FBCT images (*p* < 0.000006, *p* < 0.00001, and *p* < 0.000003, respectively). HyperSight iCBCT MAR demonstrated significantly higher percentile range across patients compared to both HyperSight iCBCT Acuros and CTsim FBCT for all real‐patient OARs (*p* < 0.0003 and *p* < 0.0001, respectively), indicating worse HU uniformity. However, this was not observed in the phantom brain analysis, where the PR values of both HyperSight images were comparable (PR_HSAcuros _= 53 HU, PR_HSMAR _= 53 HU, PR_FBCT _= 37 HU).

### 3.2 Image‐based metrics

**FIGURE 6:**
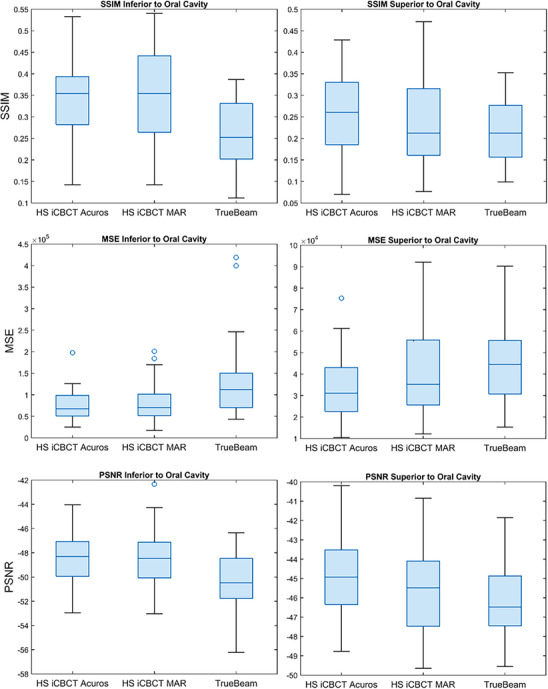
The structural similarity index measure (SSIM), mean‐square error (MSE) and peak signal‐to‐noise ratio (PSNR) values computed with the CTsim FBCT images as baseline for five slices inferior to the oral cavity five slices superior to the oral cavity.

The SSIM, MSE, and PSNR metrics for all CBCT types computed relative to CTsim FBCT are shown in Figure 6. For the inferior region, defined as the five slices below the bottom of the oral cavity, the HyperSight iCBCT Acuros and HyperSight iCBCT MAR images demonstrated significantly higher SSIM and PSNR values, and significantly lower MSE values, compared to TrueBeam (*p* < 0.02). For the superior region, defined as the five slices above the top of the oral cavity, HyperSight iCBCT Acuros achieved significantly higher SSIM values than TrueBeam, as well as significantly higher PSNR and lower MSE values than both HyperSight iCBCT MAR and TrueBeam (*p* < 0.04).

## 4 DISCUSSION

HyperSight CBCT images acquired during daily treatment on the Ethos Radiotherapy System have the potential to be used for dosimetric planning or re‐planning. This capability can minimize the need for additional CTsim imaging, reduce the time and resources associated with re‐planning, and ultimately improve the effectiveness of treatment. Adaptive re‐planning is especially relevant for head and neck patients, who are known to be susceptible to weight loss and therefore require re‐planning more frequently.^3^, ^5^, ^6^ Head and neck patients also often encounter issues related to metal artifacts caused by high‐density materials in their oral cavity, such as fillings or implants.^22^ These artifacts can obstruct the visualization of target volumes and OARs, compromise dose calculations and significantly degrade image quality, also limiting the effectiveness of image‐guided radiation therapy for patient positioning.

This work presents the first comparative investigation of HyperSight image quality conducted on a large‐population head and neck patient dataset, as a subset of a broader study that also examines dosimetric calculation accuracy, treatment planning capabilities, and adaptive performance on the Ethos platform. Previous image quality assessments have been conducted on HyperSight images of phantoms^29^, ^30^ however, real‐world patient clinical data offers distinct advantages including capturing complex structures compositions in the anatomy, patient movement during image acquisition and more realistic effects from artifacts. Furthermore, patient data provides a diversity of clinical presentations, enabling a more comprehensive and realistic assessment compared to phantom studies. A breath hold imaging study conducted by Robar et al.^23^ assessed the image quality of HyperSight images in various anatomical sites including liver, lung, breast, abdomen, chest wall, and pancreatic regions. However, evaluation of the head and neck region or artifacts associated with dental work was outside the scope of that work. Consequently, evaluation of that study did not include iCBCT MAR reconstruction mode. A study by Zhao et al.^26^ quantitatively assessed image quality metrics using phantoms, demonstrating substantial improvements in image contrast, HU consistency, and noise levels with HyperSight CBCT compared to TrueBeam CBCT. For patients with head and neck cancer as well as those with dental fillings and prostheses, only qualitative image evaluations were performed, which suggested improved image quality with HyperSight compared to standard simulation CTs. Our work builds on those findings by providing a quantitative assessment of patient images, addressing the gap left by previous evaluations.

Various image quality metrics including the artifact index, percentile range, HU accuracy and contrast were evaluated for images acquired on the state‐of‐the‐art HyperSight CBCT reconstructed with iCBCT Acuros and iCBCT MAR. These metrics were compared with those from CTsim FBCT, which served as the reference baseline, and TrueBeam CBCT.MAR algorithms are available on some CT simulators and previous studies evaluating Smart MAR on GE Optima FBCT have reported significant improvements in HU accuracy, anatomical visibility, and radiotherapy dose calculation accuracy.^34^, ^35^, ^36^ A limitation of this study is that our CTSim FBCT did not offer a MAR reconstruction option. The FBCT images in this study were reconstructed without MAR, aligning with standard clinical practice at our institution, despite 80% of patients exhibiting metal‐induced artifacts. These artifacts could compromise the accuracy of HU comparisons. However, to mitigate this issue, the slices used for quantitative analysis were carefully reviewed to ensure that the ROIs were not directly affected by artifacts on the same slice. Future studies could explore the differences in quality of images acquired on a CTsim FBCT equipped with MAR reconstruction, and HyperSight iCBCT MAR.

A significant reduction in metal artifact was observed in the oral cavity for HyperSight iCBCT MAR compared to all other imaging modalities, while HyperSight iCBCT Acuros was comparable to CTsim FBCT. Seventeen patients had a PTV located in regions affected by significant metal artifacts, compromising the visibility and HU accuracy of the target. Our results highlight how MAR effectively addresses challenges associated with metal artifacts, particularly in cases where targets and critical organs are located close to the implant. In this study, the artifact index is defined using a background ROI within the same OAR, selected from a layer with low standard deviation. Unlike in phantom studies^29^, ^30^ imaging patients in the absence of their existing implants is not feasible, making it more challenging to identify a background ROI free from the effects of metal artifacts. This may potentially impact the accuracy of the artifact index metric.

The HU measurements from the HyperSight images were systematically lower compared to baseline, while TrueBeam images exhibited higher HU measurements. This is contrary to the findings from Robar et al.^23^ where the HU measurements were lower for TrueBeam compared to CTsim. However, this discrepancy could be attributed to differences in the imaging protocols used. For all OARs, the HU and contrast values across patients were significantly closer to baseline in the HyperSight images compared to TrueBeam, except for HU measurements in the parotid glands, which may be attributed to the higher HU variation observed in the TrueBeam images. HyperSight iCBCT MAR showed the highest soft‐tissue HU accuracy across imaging modalities with an average MAD of 14 HU. This accuracy could translate into CBCT‐based dose calculation differences of less than 1% in the head and neck region.^37^ Although bone HU accuracy was also higher for the HyperSight images compared to TrueBeam, the difference was not statistically significant due to the high variability observed in the HyperSight images.

The percentile range was used to evaluate image non‐uniformity in the oral cavity, brain, left and right eyes and brainstem for both of the HyperSight images and the FBCT images. This metric was only applicable to the oral cavity and brainstem in the TrueBeam images due to the limited field of view. HyperSight iCBCT MAR displayed significantly higher percentile range values in all OARs, indicating worse HU uniformity compared to both HyperSight iCBCT Acuros and CTsim FBCT, although still better than TrueBeam. The percentile range differences are visually apparent in the top row images of Figure 1d, where the brain appears smoother in the HyperSight iCBCT Acuros image and increased noise is observed in the HyperSight iCBCT MAR image. Given the brain's proximity to the top of the image, we aimed to exclude the image periphery when selecting the brain ROI for computing the percentile range to avoid the cone beam effect. Similarly, for accurate dose calculation, it is important to either establish boundaries from the field edges or extend the scan well beyond the planned treatment and contour areas. The percentile range in the brain of an anthropomorphic phantom was evaluated to provide a more controlled and ideal scenario for assessing HU uniformity. While no decrease in HU uniformity was observed in MAR images compared to Acuros (likely due to the simplicity of the phantom composition), the CTsim FBCT images exhibited greater HU uniformity than both HyperSight modalities. This observation is consistent with the results from real‐patient images, while ensuring that HU variability is not influenced by anatomical tissue density differences.

SSIM, PSNR, and MSE were used to assess image quality by comparing all images to the CTsim FBCT as the baseline ground truth. An SSIM close to one, a higher PSNR, and a lower MSE all indicate greater similarity to the reference image, reflecting image quality that is comparable to FBCT. In the slices inferior to the oral cavity, SSIM, MSE, and PSNR metrics demonstrated significantly higher similarity to baseline for both HyperSight reconstructions compared to TrueBeam. In the superior region, HyperSight iCBCT Acuros outperformed both TrueBeam and HyperSight MAR. This area contains a mix of high‐ and low‐density tissues (bone and air), where MAR reconstruction may introduce inaccuracies by misclassifying bone as metal. These results, in addition to the HU uniformity analysis, suggest that while MAR improves image quality in the presence of artifact‐producing metal implants, it may not always be the optimal default imaging mode. The iCBCT Acuros algorithm may be preferred when artifact reduction is not a concern.

One limitation of this study is the timing of image acquisition. The HyperSight CBCTs were obtained at the same session as the FBCT, ensuring minimal anatomical variation between scans. In contrast, the TrueBeam CBCTs were acquired at the first treatment fraction, introducing potential time‐dependent anatomical changes that may have affected the comparison. Some image differences were observed in skin folds behind the patient's neck, likely due to mask setup variability, or in shoulder positioning. Although these differences were also present in the HyperSight images, the specific regions of interest analyzed were unaffected after rigid registration, as they were located away from superficial variation.

The images in this study were acquired with different pixel sizes (FBCT: 1.27 mm, TrueBeam CBCT: 0.51–0.91 mm, HyperSight CBCT: 1.37 mm), which likely contributed to the variations observed in image quality metrics. Larger pixels in HyperSight CBCT and FBCT reduce noise and improve image uniformity and HU stability in homogeneous regions but may compromise resolution and fine detail. Conversely, the smaller pixels in TrueBeam CBCT allow higher spatial resolution, but are more sensitive to noise and artifacts, which can degrade HU accuracy and increase the artifact index. In a study by Yoo and Yin^38^ HU differences between planning CT and conventional CBCT for an inhomogeneous phantom ranged from 50 to 200 HU, leading to dose calculation discrepancies of up to 3%. Similarly, Ding et al.^39^ reported HU discrepancies of up to 100 HU between CT and CBCT images, with resulting dose calculation errors typically within 1%–3% for both phantom and patient IMRT plans. Barateau et al.^40^ observed differences of up to 900 HU in the electron density calibration curves across different phantoms. Despite these large differences, the dose calculation discrepancies in an anthropomorphic phantom remained within 3% across head and neck and pelvic regions regardless of the calibration curve used. These studies demonstrate that although HU uncertainties in CBCT imaging can be substantial, their impact on dose calculations are generally low. Even though the HU differences observed in our study for HyperSight images were significantly lower (<15 HU) than for conventional CBCT systems, dose calculation accuracy alone does not fully determine the clinical adequacy of an image for treatment planning. Clear visualization of the target and surrounding organs is essential since the treatment plan is guided by their contours. In contrast to the mentioned studies, our work incorporates quantitative image quality metrics, including artifact index and contrast to objectively assess image clarity.

This study highlights HyperSight's benefits in terms of image quality over traditional on‐board imaging modalities for head and neck patients. Recent studies have begun evaluating the clinical viability of HyperSight CBCT for direct dose calculation across various treatment sites, demonstrating accuracy comparable to synthetic CTs and planning CTs.^41^, ^42^, ^43^ To confirm the suitability of HyperSight images for precise dose calculations in the head and neck region, we have initiated a follow‐up dosimetric investigation comparing forward calculated treatment plans on the images from the same patient cohort. Future research will also focus on validating these outcomes through a clinically qualitative image assessment and the delineation and verification of critical organs. These efforts are crucial for effectively translating our findings into clinical practice and integrating HyperSight into adaptive radiotherapy workflows.

## 5 CONCLUSION

The HyperSight iCBCT MAR reconstruction algorithm significantly reduced image artifacts in the oral cavity compared to HyperSight iCBCT Acuros, TrueBeam, and CTsim FBCT, offering substantial benefits for dental implant cases. Additionally, HyperSight iCBCT MAR and iCBCT Acuros images demonstrated similar soft‐tissue HU and contrast characteristics to CTsim FBCT in critical OARs within the head and neck region, supporting their use in adaptive treatment workflows where accurate image quality is essential for delineation and dose calculation, with further evaluation of dose accuracy to be presented in future work. The results from this study will support the implementation of the high‐performance Ethos system in our clinic and in the broader radiation oncology community.

## AUTHOR CONTRIBUTIONS

L.M. and A.C. contributed to the design of the study, L.B., M.R., and D.W., contributed to patient recruitment, K.Z., J.G., N.M., and D.M., contributed to data acquisition, L.W., C.A., and M.L. contributed to data processing, and A.Y., L.M., and A.C. contributed to the analysis and interpretation of data. A.Y. drafted the manuscript and all authors critically reviewed and revised it. All authors approved the final version to be published and agree to be accountable for all aspects of the work in ensuring that questions related to the accuracy or integrity of any part of the work are appropriately investigated and resolved.

## CONFLICT OF INTEREST STATEMENT

Amanda Cherpak and Lee MacDonald are members of Varian’s Intelligent Imaging Consortium (IIC) and other authors have no conflicts of interest to declare.

## Data Availability

Access to unpublished data from our study is subject to negotiation with the study sponsor, Varian Medical Systems, Inc., or as may be required by applicable law.
